# Open surgery with a lateral neck approach in cases of foreign body impaction that penetrating the neck through the esophagus: a single-center experience

**DOI:** 10.1186/s12893-024-02449-5

**Published:** 2024-05-18

**Authors:** Feng Xu, Na Shen, Danzheng Liu, Ting Zhu, Liang Xue, Xuemei Luo, Xu Zhou

**Affiliations:** 1 Zhongshan Hospital, Fudan University, Fenglin Road 180, Shanghai, 200032 China; 2 Department of Vascular Surgery, Zhongshan Hospital, Fudan University, Shanghai, China; 3 Department of Department of Thoracic Surgery, Zhongshan Hospital, Fudan University, Shanghai, China

**Keywords:** Lateral neck approach, Cervical esophagus foreign bodies impaction, Open surgery, Surgical skills, Treatment

## Abstract

**Background:**

Because the cases are quite scarce, we aimed to review cases of foreign body impaction penetrating the neck through the esophagus to analyze the characteristics of these cases. The open surgery skills of the surgeon, the treatment procedure and the surgeons’ experience in the rare diseases were analyzed.

**Methods:**

We collected and analyzed all cases from 2015–2020 in our hospital. Surgical skills and procedures for fasting and anti-infection treatment were reviewed retrospectively. Follow-up was telephone communication.

**Results:**

Our series included 15 cases. Tenderness in the pre-cervical site was a physical sign for screening. Thirteen cases underwent a lateral neck open surgery with the incision including the left side of neck and only two cases were incised from the right side of the neck. Pus was found 3 days after the impaction in one case, the shortest time observed in our series. The esophageal laceration was only sutured primarily in 5 cases (33.33%) among all fifteen cases. After sufficient drainage (average more than 9 days), antibiotic treatment and fasting (normally 2–3 weeks), patients gradually began to switch to solid foods from fluids after complete blood counts and confirmations from esophageal radiography result. No severe complications occurred, and all the patients have no swallowing dis-function and recovered well.

**Conclusion:**

Surgery should be performed as soon as possible after impaction. Lateral neck approach surgery and the therapeutic procedure described in this article are safe and effective treatments.

## Introduction

Esophageal foreign body impaction occurs often in China, particularly due to the frequent consumption of sharp-pointed food, such as meat with bones [1]. Foreign body impaction occurs relatively frequently in elderly with dentures, who often mis-swallow foreign bodies because the denture base prevents the tongue touching palate, resulting in difficulty in distinguishing the bones from the rest of the food. Typical foreign bodies commonly include fish, chicken and duck bones, jujube seeds and dentures. This often blocks the entrance to the esophagus [2]. Endoscopic removal for esophageal foreign bodies is safe, effective and convenient [3, 4]. In our hospital, most foreign bodies were removed using gastroscopy. In the past two years alone, more than 800 patients have undergone gastroscopic surgery for esophageal foreign body impaction in our hospital [5].

However, in some severe cases, sharp-pointed and slender esophageal foreign bodies pass out of the esophagus into cervical soft tissue due to attempts to relieve the impaction by ingesting more solid food after mis-swallowing. Sometimes, these foreign bodies can even pierce blood vessels and the thyroid gland [6–8]. Neck abscesses, mediastinitis, mediastinal abscesses and pseudoaneurysm may occur a few days after the impaction, all of which can be life-threatening. Therefore, traditional endoscopic surgery cannot find the foreign body in the esophagus lumen. Open surgery is the only way to remove foreign bodies and treat complications. However, to date, the studies about open surgery and treatment procedures have been rather scarce.

In this study, we aim to analysis and study the clinical characteristics of the cases of foreign bodies impaction in the cervical soft tissue penetrating through the esophagus which were treated by open neck surgery. Cases treated in our hospital between 2015 and 2020 were reviewed according to the medical records retrospectively with patient clinical characteristics, surgical approach, treatment procedure and surgeon experience.

## Materials and methods

### Patient populations

This is a retrospective narrative study. Through medical records, we collected cases of foreign bodies in the esophageal entrance visited in our hospital between 2015 and 2020 (Table 1) in which the presence of cervical esophageal foreign bodies was first confirmed using computed tomography (CT) [9]. Cases in which the foreign body protruded from the esophagus into the cervical soft tissue space and could not be removed with gastroscopy and cases of severe cervical infection due to large esophagus perforation were included in our study. All the cases that met the criteria are described in the study. This study was approved by our hospital ethics committee, number B2022-179.

**Table 1:** The clinical features of the cases which were treated by neck open surgery between 2015–2020. ^a^The length is the distance from the incisor teeth in the esophagus

	**Name**	**Gender**	**Age**	**Foreign bodies**	**Time to presentation**	**Site**^**a**^	**Side of incision**	**Fever or no**	**Pus or no**	**Oesophagus was** **sutured primarily**	**Others**
1	LFX	F	68	Jujube seed	4 days	15 cm	left	fever	Pus	No	No
2	YCF	F	67	Fish bone	14 days	Upper esophagus	right	fever	Pus	No	Left subclavian pseudoaneurysm
3	JQZ	F	75	Fish bone	9 h	20 cm	left	fever	No	No	No
4	JYF	M	49	Fish bone	9 days	20 cm	left	fever	Pus	No	No
5	GYP	M	65	Denture	4 days	entrance	left	no	No	No	No
6	LW	M	50	Chick bone	15 days	Upper esophagus	Left	No	No	Yes	No
7	PYJ	M	43	Fish bone	7 days	T2,3	left	no	No	No	No
8	WGH	F	66	Fish bone	10 days	Entrance	left	fever	Abscess cavity	No	No
9	WJH	F	66	Fish bone	19 days	Upper esophagus	Right	fever	Abscess cavity	No	No
10	TQ	F	32	Iron wire	1 day	16 cm	right	fever	No	Yes	No
11	DCJ	F	41	Fish bone	7 days	No data	Collar incision	No	Pus	No	Common caroid pseudoaneurysm
12	ZJP	M	63	Chicken bone	3 days	Cervical esophagus	left	fever	Pus	No	No
13	MJC	M	34	No data	5 days or more	T1 level	left	fever	Pus	No	No
14	JXM	F	73	Iron wire	4 days	Upper esophagus	left	fever	Pus	Yes	No
15	LJJ	M	37	Fish bone	4 days	Cervical oesopgagus	left	No	No	Yes	No

### Surgical procedure

Surgical management should be carried out as early as possible for perforating foreign body impacts in esophagus. The patients were placed in a supine position with their neck extended and their head tilted to the healthy side while under general anesthesia. A vertical incision was usually made at the level of the foreign body, commonly along the front edge of the sternocleidomastoid muscle and ended approximately two-fingers-width above the superior sternal fossa. The length of the incision was adjusted based on the position of the foreign body in the neck. The platysma muscle layer was incised, exposing the anterior edge of the sternocleidomastoid muscle, which was retracted posteriorly, and then the strap muscles were vertically divided. The muscle fibers were retracted forward and backward respectively, and the thyroid lobe and carotid sheath were exposed. The scapula hyoid muscle was severed if needed, after which the thyroid gland lobe was retracted medially if the foreign body was behind the thyroid lobe and the cervical sheath was retracted laterally to expose the lateral edge of the esophagus. When the patient’s head is turned to the healthy side, the esophagus will be slightly biased in this direction. The recurrent laryngeal nerve was identified and deliberately protected in the sulcus of the esophageal and trachea along the tracks; identification of these structures was crucial. Rigid sharp foreign bodies were often found along the edge of the esophagus and could be palpated by with fingers in some cases. The space around esophagus was explored and separated along its length, and a syringe was sometimes useful for locating pus. After this, the foreign body was located in the abscess cavity. Sometimes, it is difficult to locate the foreign body that it is too small or wide tissue fibrosis due to infection. Bedside X ray can help to locate the foreign body if required. If the presence of an upper mediastinal abscess was confirmed by preoperative CT, the upper mediastinum was explored was explored downwards carefully and gently with fingers and forceps along the sulcus of the esophagus and trachea, and the pus was drained sufficiently. The cavity was then washed with a large amount of diluted povidone iodine solution and normal saline. After the esophageal perforation was found, it was usually sutured; if no esophageal mucosa edema was present, the esophageal mucosa layer could usually be primary closed, followed by the muscle layer. If the esophageal mucosa was edematous or the laceration was too large, sutures had to be performed during a second surgery if the perforation can’t be healed by itself. Finally, conventional silica gel tubes were placed near the laceration for drainage. Sometimes a double-lumen cannula was useful for draining super-mediastinum abscesses.

### Medical treatment

Sufficient amounts of carbapenems (1 g, q8h, ivgtt normal) and metronidazole (1 g, q12h, ivgtt normal) according to the patient’s weight were administered intraoperatively and postoperatively for antibacterial treatment. Nasogastric tube intake, enterostomy or total parenteral nutrition was provided to meet the patients’ daily energy requirements. The drainage tube was rinsed daily if a double-lumen cannula existed, and negative pressure suction was continued, until the drainage fluid gradually cleared and stopped. The routine silica gel tube could usually be removed when less than 10 ml of drainage fluid and complete blood counts showed no obvious bacterial infection. After no exudation of barium was confirmed using esophageal radiography, the patients were permitted to consume sterile water orally for several days. After additional normal complete blood count results were obtained, patients were allowed to consume fluids and a gradual transition from liquids to semi-fluid and solid diets was recommended.

### Case 1

**Fig.1:**
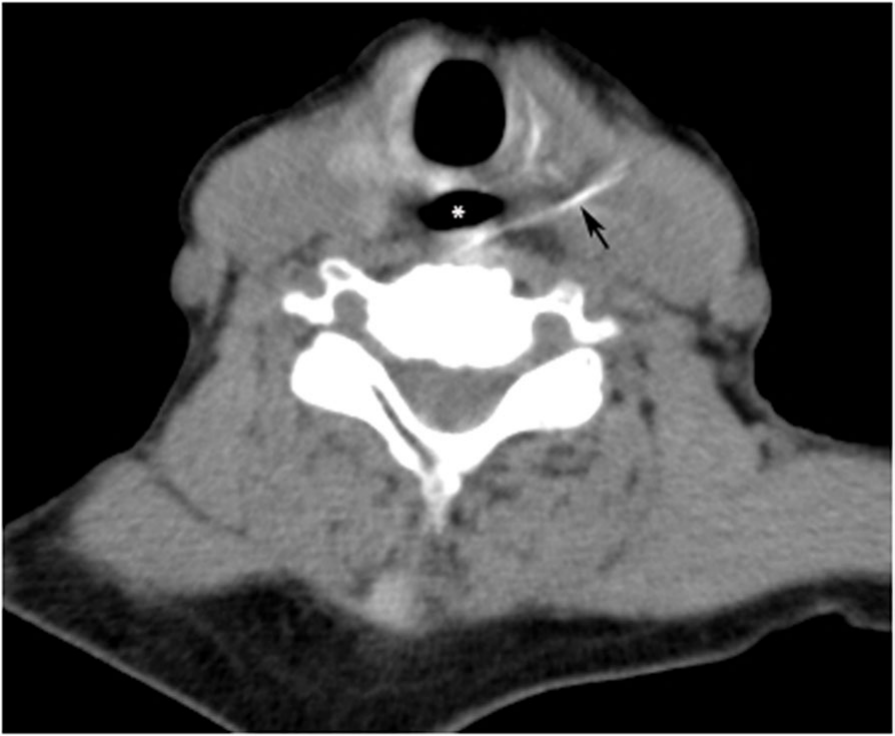
A fish bone penetrating the left neck through the esophagus in case 1. It could not be located in the esophageal lumen through endoscopy and was located in the space between laryngeal intrinsic muscles and cervical artery sheath at C7 level. Black arrow: fishbone. White star: esophagus

**Fig. 2:**
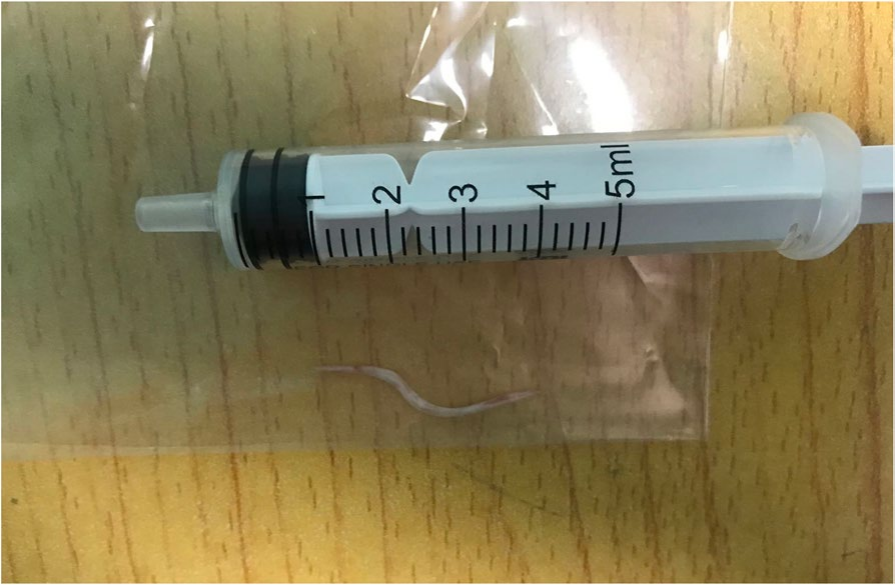
It showed the fishbone that migrated into the cervical muscles adjacent to the esophagus in case 1

JQZ (patient No. 3) was a 75-years-old female patient who presented with pain during swallowing for several hours after mis-swallowing fish bones. Esophageal CT scans showed that the foreign body had penetrated the left side of the esophagus at C7 level and entered the neck muscles space (Fig. 1). We incised the lateral neck under general anesthesia and found the fishbone in the para-esophageal space, removed it, found and sutured the esophageal laceration, and closed the incision in layers after leaving a drainage tube around esophagus (Fig. 2). The patient was fed via nasogastric tube postoperatively and given antibiotics against bacterial infection. She was transferred from our hospital to a community hospital on the 5th day after surgery with nasogastric tube and drainage tube.

### Case 2

**Fig. 3:**
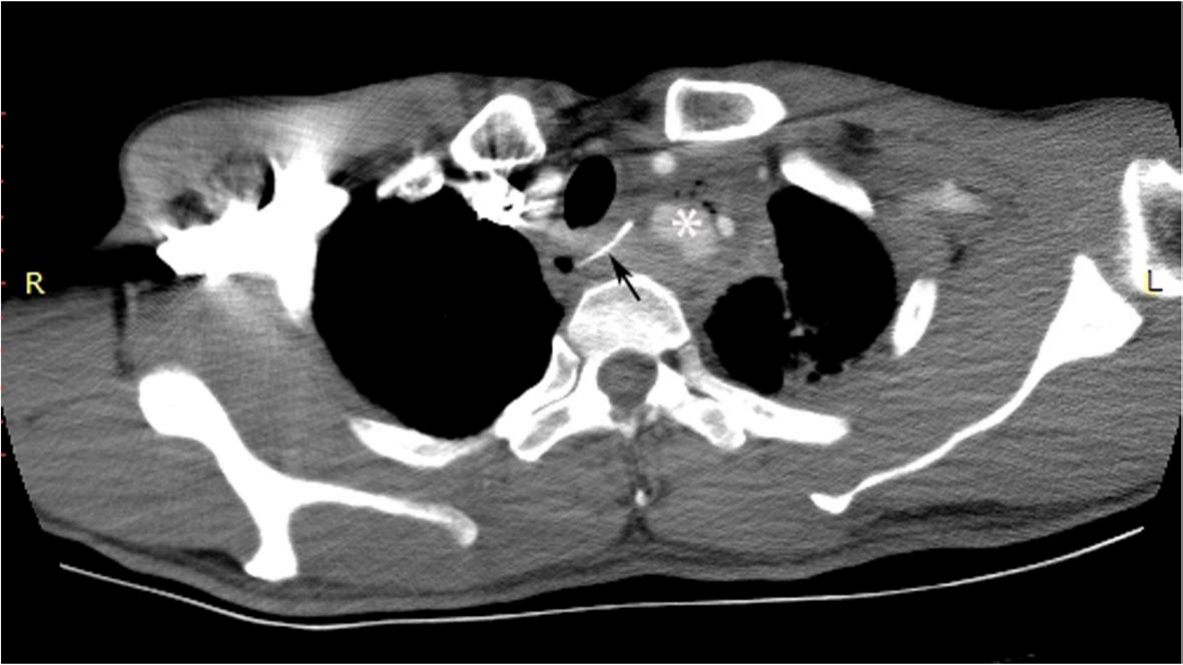
Enhanced computed tomography showed a fish bone that had penetrated the left superior mediastinum through the esophagus. An aneurysmal hematoma had formed because the left subclavian artery was pierced. Exudation is present in the mediastinum around esophagus and left pleural cavity. Black arrow: fish bone; White star: aneurysmal hematoma enhanced by contrast medium

GYP (patient No. 5), a 49-year-old man, choked on a fish bone at the beginning of the esophagus for 9 days. He ignored swallowing pain for several days but eventually sought help because he developed fever, chills, and weakness. A CT scan showed that the fish bone was protruding into the superior mediastinum and had formed a mediastinal abscess and left subclavian artery aneurysmal hematoma (Fig. 3).

First, an emergency surgery was performed on the blood vessel. The patient was placed in a supine position and the puncture site was prepared with sterile draping. The right femoral artery was punctured under local anesthesia (1% lidocaine, about 5 ml, subcutaneous injection), and a 5F vascular sheath was inserted into the puncture site. Arteriography was performed using a 5F angiographic catheter in the aortic arch and subclavian artery to confirm the location of the pseudoaneurysm. A 12F sheath was exchanged in the puncture access. A super stiff guidewire was introduced across the lesion. A Viabahn stent was deployed at the position of the lesion and angiography revealed that the pseudoaneurysm was well disappeared.

With the patient under general anesthesia, we then found the fish bone in the esophagus 20 cm away from the incisors teeth and removed it using a gastroscope. The abscess near the esophagus in the superior mediastinum was then drained and the necrotic tissue around it was cleared during the open left cervical open surgery. The esophageal perforation was un-sutured. Because other patients were waiting for surgery, after two days, this patient was safely transferred to the community hospital with a drainage tube and under fasting conditions.

### Case 3

WJH (patient No. 9) was a 66-year-old woman who presented with retrosternal pain and a fever of 39.2℃ and mis-swallowed a fishbone 13 days ago. The patient’s body temperature rose to 44℃ despite undergoing a six-day antibiotic course. We performed right cervical surgery with an incision along the right anterior edge of the sternocleidomastoid muscle, explored the fibrous thick-walled abscess cavity along the right side of esophagus, and removed a 4-cm-long fishbone finally. The operational field was then drained and flushed. After the drainage pipe was placed, the incision was sutured layer by layer. The patient received the same strategy of fasting and antibiotic therapy as with in previous cases, then recovered.

## Results

### Patient characteristics

Among the 15 patients analyzed, 7 were male (46.67%) and 8 were female (53.33%) with a median age of 56 (range, 32–75 years). Patients endured painful swallowing for a period of 9 h to 19 days (median 4 days) before open cervical surgery. In our series, it is 13 cases whose foreign body or the abscess cavity are in the left cervical side (86.67%), and only 2 cases are in the right (13.33%). All patients showed tenderness above the upper suprasternal fossa (Table 1).

### Surgical procedure

The delay in pus development was correlated with the size of the perforation. The larger the size of the esophageal laceration, the sooner pus occurs. And pus was found in a minimum of 3 days after choking in our case. Left cervical incisions were performed in twelve cases (80.00%) according to the location of the foreign body or abscesses cavity while right cervical incisions were only performed in two (13.33%), the cervical collar incision in one case (6.67%) (Table 1).

In our 15 cases, esophageal laceration was sutured in 5 cases in primary surgery (33.33%), in the other 10 cases (66.67%), it was un-sutured due to edema mucosa and healed itself. No cases of muscle repair were recorded in our cases. After recovery, all the patients can swallow normally without esophagus stenosis.

There are two cases (13.33%) related with pseudoaneurysm and vascular surgery, one is on the common carotid artery, and the other on the subclavian artery. Stent was placed to avoid blood vessel rupture.

Two cases (13.33%) had large abscess cavities and severe tissue fibrosis. They lead problems in locating foreign bodies, esophagus and the common carotid artery and senior surgeons attended the surgery. Their medical history is 10 and 19 days, respectively. Regular silica pipe was put in the incision in all of cases and double-lumen cannula were used for superior mediastina drainage in two cases (13.33%). The average withdrawal time was no less than 9 days (8.5 days) in all 15 cases (because some cases were transferred to community hospitals with drainage pipes before recovery and the medical records could not be obtained) and 2–3 weeks was common.

### Medical treatment

Intraoperative and postoperative antibiotic drugs typically included carbapenem and Ornidazole in all cases. Antibiotics were administered and not stopped until patients can consume a solids diet by mouth.

The drainage tube in the neck was removed when the volume of fluid was less than 10 ml in all cases and the CT scan showed that the inflammatory area around esophagus had narrowed. Before the patients were allowed to eat by mouth, they consumed food through a nasogastric tube or were supported entirely by intravenous nutrition, typically for approximately two weeks, or three weeks in some cases. Of note, in patient No. 1 (Table 1), it took two months for the esophagus perforation to heal itself with feeding enterostomy because the laceration of the esophagus is more than 3 cm long and cannot be suture primarily due to edematous mucosa. Then food could be taken by mouth, transitioning from fluids to solids gradually over several days, accompanied by a complete blood count every 3 days. There were no serious postoperative complications in this series.

## Discussion

The clinical presentations were similar in that the patients commonly complained of dysphagia and odynophagia after mis-swallowing foreign bodies. CT is a useful method for diagnosing foreign body impaction in the esophagus [4]. More image informatiom, such as the location of the foreign body in the neck tissues, the thickened of esophagus, extraluminal air in the soft tissue of the neck due to the esophageal perforation and abscess cavities adjacent to the esophagus, and even the arterial aneurysmal if present, can be visualized on CT. Information on the relationship between the foreign body and important cervical structures is important for open surgery. Especially, tenderness was most obvious at the pre-cervical sites, and above the upper suprasternal fossa. It could be a screening physical sign of cervical esophageal foreign body impaction.

The surgery was an emergency. Complications of foreign body impaction are directly related to retention time in the esophagus. If the foreign bodies are retained for at least 3 days, pus will appear, followed in turn by an abscess cavity wrapped in fibrous tissue, making it more difficult to be found during open surgery. This increases the possibility of infection and severe hemorrhage. Aneurysmal hematoma and mediastinum abscess were not uncommon complications. Therefore, the operation should be performed as soon as earlier.

In cases of a longer medical history, fibrous connective tissue surrounding the abscess may be present. The fibrous wall of the abscess was hard and fused with the surrounding tissue structure; identification of the foreign body was difficult. The thyroid cartilage was useful for locating the trachea and trapped muscle, and the cervical vertebra was useful for locating the esophagus. The bony structure, such as cervical centrum and thyroid cartilage, is useful for locating the key structures.

Perforation of the cervical esophagus will lead to abscesses in the tissues surrounding the esophagus and even the upper mediastinum. Infection dissemination is limited because the esophagus is attached to the prevertebral fascia and the spread of infection to the mediastinum is slow. Regardless, such infections are usually mild [10] and can be treated with sufficient flushing, drainage and broad-spectrum antibiotics. Sometimes the foreign body may perforate the carotid artery and cervical veins and cause massive bleeding. It should first be treated with vascular surgery and open cervical surgery immediately.

Adequate flushing and drainage are crucial to accelerate the recovery after surgery. A two-lumen tube is sometimes required to treat abscesses in the superior-mediastinum. One lumen for flushing and the other for draining. Certain procedures for oral intake were then followed. This procedure is relatively safe, no cases returned to the hospital due to postoperative reinfection around the esophagus after transition to a normal diet in our cases.

We should suture the esophagus laceration in every case in theory. In practical, the mucosa is too edematous to be sutured due to inflammation and infection. The mucosa under contaminated condition was edematous and cannot be sutured which often happened 24 h after damage. Additionally, the laceration was usually small. So, they are common un-sutured. Most of them might heal themselves after drainage and anti-infection treatment. Note that in the patient No. 1 (Table 1), the laceration of the esophagus was too long of 3 cm to be sutured. Adequate drainage and enterostomy were performed to provide nutritional support and the patient still recovered well, starting on an oral diet after two months. Larger lacerations should perhaps also be repaired in secondary surgery or with muscles adjacent to the esophagus or free colon.

Among the 15 cases, 12 underwent incision on the left side of the neck. Foreign bodies appear to easily enter the left cervical side, which may be related to the anatomical features of the cervical esophagus. Because this organ, passes through the front-left side of the vertebral column, foreign bodies are prone to penetrate the retroesophageal space from the left side of the esophagus, and left cervical incisions are more convenient for exposing the esophagus, the foreign body, and the abscess. No severe intra- and postoperative complications occurred in these cases. Although the left recurrent laryngeal nerve is closer to the midline than the right nerve [11], no additional neurological damage was caused by the incision of the left side of the neck.

Some authors that consider posterior incision along the sternocleidomastoid muscle to also be feasible [12]; however, we incised the neck along the anterior border of the sternocleidomastoid because it is closer to the esophageal perforation. The incision is safe and convenient if the anatomy of the thyroid is familiar, and in our study, no complications of the blood vessels or nerves occurred in the cases that underwent the anterior incision.

More than 800 cases of esophageal foreign body impaction were observed in our hospital in one year, most of which involved choking on fish bones, jujube seeds, chicken and duck bones and dentures, which can be removed with gastroscopy. Relatively few cases require surgical incision due to the protrusion of esophageal foreign bodies into the neck space. But in those cases, an earlier intervention is best. Medical treatment often lasts more than one week, and a period of two to three weeks is ideal. Practical application of high-level antibiotics of sufficient dosage has been shown to be necessary. No recurrent infectious were observed in our series.

Surgery with a lateral neck approach can often be used to treat cases of mis-swallowed foreign bodies that protrude from the esophagus into the neck, in addition to abscesses of the neck and super mediastinum. The procedure introduced in this study is a safe and feasible method for treating foreign body impaction.

## Data Availability

All data generated or analyzed during this study are included in this published article and its supplementary information files.
